# Evaluation of toxicity of the mycotoxin citrinin using yeast ORF DNA microarray and Oligo DNA microarray

**DOI:** 10.1186/1471-2164-8-95

**Published:** 2007-04-05

**Authors:** Hitoshi Iwahashi, Emiko Kitagawa, Yoshiteru Suzuki, Youji Ueda, Yo-hei Ishizawa, Hitoshi Nobumasa, Yoshihide Kuboki, Hiroshi Hosoda, Yumiko Iwahashi

**Affiliations:** 1 Human Stress Signal Research Center, National Institute of Industrial Science and Technology, AIST, Tsukuba West, Onogawa Tsukuba, Ibaraki 305-8569, Japan; 2 DNA Chip Research Inc. 1-1-43, Suehiro-cho, Tsurumi-ku, Yokohama, Kanagawa, 230-0045, Japan; 3 Toray Industries, Inc, 1111, Tebiro, Kamakura,248-8555, Kanagawa, Japan; 4 Toray Industries, Inc, 1-1, Nihonbashi-Muromachi 2-chome, Chuo-ku, Tokyo 103-8666, Japan; 5 National Food Research Institute, NFRI, 2-1-12 Kannondai, Tsukuba, ibaraki, 205-8642, Japan

## Abstract

**Background:**

Mycotoxins are fungal secondary metabolites commonly present in feed and food, and are widely regarded as hazardous contaminants. Citrinin, one of the very well known mycotoxins that was first isolated from *Penicillium citrinum*, is produced by more than 10 kinds of fungi, and is possibly spread all over the world. However, the information on the action mechanism of the toxin is limited. Thus, we investigated the citrinin-induced genomic response for evaluating its toxicity.

**Results:**

Citrinin inhibited growth of yeast cells at a concentration higher than 100 ppm. We monitored the citrinin-induced mRNA expression profiles in yeast using the ORF DNA microarray and Oligo DNA microarray, and the expression profiles were compared with those of the other stress-inducing agents. Results obtained from both microarray experiments clustered together, but were different from those of the mycotoxin patulin. The oxidative stress response genes – *AADs*, *FLR1*, *OYE3*, *GRE2*, and *MET17 *– were significantly induced. In the functional category, expression of genes involved in "metabolism", "cell rescue, defense and virulence", and "energy" were significantly activated. In the category of "metabolism", genes involved in the glutathione synthesis pathway were activated, and in the category of "cell rescue, defense and virulence", the ABC transporter genes were induced. To alleviate the induced stress, these cells might pump out the citrinin after modification with glutathione. While, the citrinin treatment did not induce the genes involved in the DNA repair.

**Conclusion:**

Results from both microarray studies suggest that citrinin treatment induced oxidative stress in yeast cells. The genotoxicity was less severe than the patulin, suggesting that citrinin is less toxic than patulin. The reproducibility of the expression profiles was much better with the Oligo DNA microarray. However, the Oligo DNA microarray did not completely overcome cross hybridization.

## Background

Mycotoxins are fungal secondary metabolites commonly present in the feed and food, and are widely considered as hazardous contaminants. However, the toxicity of these natural chemicals are not properly evaluated because of the difficulties in isolating these chemicals and also because of the lack of interests as they have no industrial applications. The costs for producing the pure mycotoxins are the biggest obstacle in their evaluation process. On the other hand, development of analytical methods are needed to identify new mycotoxins, to fight against the spreading toxins, and also to meet the growing demands for the toxicological studies.

**Figure 1:**
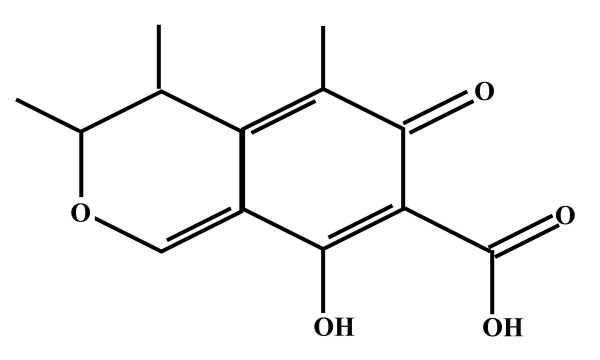
Chemical structure of citrinin.

Citrinin [518-75-2], 4,6-dihydro-8-hydroxy-3,4,5-trimethyl-6-oxo-3H-2-benzopyran-7-crboxylic acid (Figure 1), which was first isolated from *Penicillium citrinum *[1], is produced by more than 10 kinds of fungi [1]. Citrinin is the one of the well-known mycotoxins, which is possibly spread all over the world. Although citrinin is one of the well-characterized mycotoxins, information on its mechanism of toxic action is limited. Clinically, citrinin was shown to cause renal disease in poultry, pigs, dogs and rats [2,3]. The electron transport system of the kidney and liver mitochondria were considered as the target of the toxic action of citrinin [4].

The availability of yeast DNA microarrays provides the possibility of monitoring gene expression levels as a function of toxin exposure, and consequently, provides a mean to determine the mechanism of toxicity [5,6]. The essential features of this yeast system are the small volume of yeast culture required for the analysis, high reproducibility of the expression profiles and availability of the massive functional information of genes on DNA microarray [7,8]. For example, cadmium treatment was found to induce yeast genes involved in the sulfur amino acid metabolism, oxidative stress response, and heat shock response [6]. This expression pattern of induced genes was in agreement with many previous studies [6]. We applied this system to evaluate the action mechanism of patulin, one of the most potent mycotoxins, and found that patulin targets proteins and possibly DNA [7]. Our results suggested that patulin probably acts as a mutagen [7].

In this report, we studied the toxicity of citrinin to yeast cells using the traditional ORF (Open Reading Frame) DNA microarray [6] and Oligo (Oligo-nucleotide) DNA microarray systems [9]. Results from both microarray studies suggested that the oxidative stress was the main cause for toxicity, but this oxidative stress did not lead to any DNA damage. This observation was different from what was found with another mycotoxin patulin [7]. To detoxify against the citrinin, the yeast cells mainly used glutathione modification and pumped out the toxin using transporters. We have also discussed how the two DNA microarrays were adapted for evaluating the mycotoxin action.

## Results

### Conditions for the citrinin treatment

**Figure 2:**
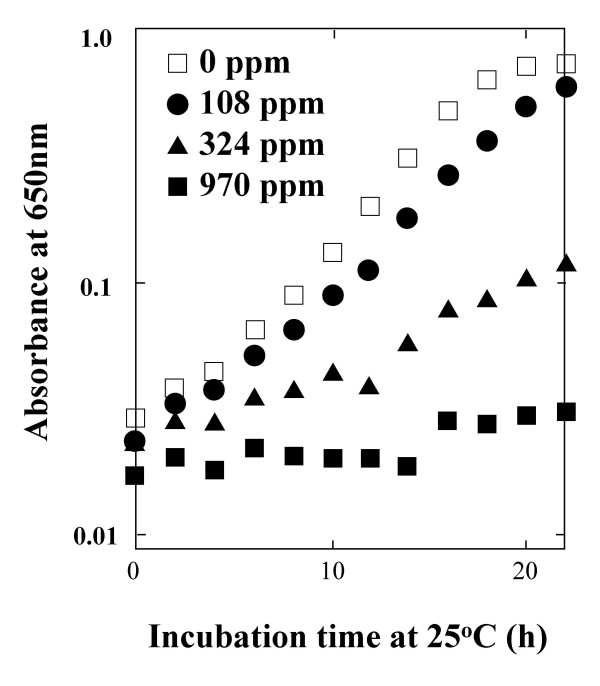
**Effect of citrinin on yeast growth**. Citrinin dissolved in DMSO at a concentration of 20000 ppm was added to the YPD medium to achieve the indicated concentration. The stock solution was added directly to the yeast cells grown for 2–3 days such that they were diluted more than 100-fold.

As a first step, we characterized the effect of citrinin on yeast growth because without any biological or physiological characterization we will not be able to prove that the induction or repression of specific genes is due to the treatment. Lack of growth inhibition would merely indicate that the conditions used for the study did not cause any cellular stress. Figure 2 shows yeast growth as a function of different concentrations of citrinin. As shown, we observed growth inhibition at concentrations greater than 108 ppm, and at 970 ppm of citrinin there was no growth. Based on this dose-response analysis, 300 ppm of citrinin was chosen for subsequent experiments, as this concentration was found to be inhibitory to non-lethal growth (data not shown). This concentration citrinin is slightly higher than that was used for the patulin treatment [7], and citrinin may be less toxic to yeast cells.

### Overview of citrinin induced and repressed genes through ORF DNA microarray and Oligo DNA microarray

**Figure 3:**
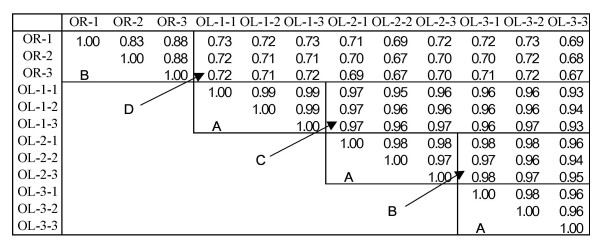
**Correlation factors among the different experiments (same conditions but different treatments)**. A, Different sheets of microarray. B, Different citrinin treatment. C. Different labeling (dye swap), D, Different types of microarray. Dye swap was carried out with the OL-1-1, OL-1-2 and OL-1-3 sheets.

From three independent citrinin treatment experiments, we obtained 12 sheets of DNA microarray results. Three sheets (OR-1, OR-2, OR-3 in Figure 3) were from the ORF DNA microarray, one from each citrinin treatment. For the Oligo DNA microarray, we performed three hybridizations for each experiment and obtained 9 sheets of data (OL-1-1, OL-1-2, OL-1-3, OL-2-1, OL-2-2, OL-2-3, OL-3-1, OL-3-2, OL-3-3 in Figure 3), including dye swap for the OL-1-1, OL-1-2, and OL-1-3 sheets. From the microarray data (Figure 3) we calculated the correlation factors to determine the reproducibility between the different hybridization conditions (region A in Figure 3), citrinin treatment (region B of Figure 3), dye swap (region C of Figure 3), and DNA microarray (region D in Figure 3). The correlation factors for the ORF DNA microarray were from 0.83 to 0.88. For the Oligo DNA microarrays, the correlation factors were from 0.93 to 0.99 for 9 sheets, and from 0.96 to 0.99 for the same source of total RNA (Figure 3). The correlation factors between the ORF DNA microarray and Oligo DNA microarray showed relatively low correlation factors (0.67–0.73) than those among the same type of DNA microarray. These results suggest that the reproducibility of the Oligo DNA microarray is better than those of the ORF DNA microarray (Region B in Figure 3).

From the ORF DNA microarray, we obtained 5,928 ORFs exhibiting intensities over the cut-off value at least in one experiment. Among these ORFs, 155 ORFs showed more than two times higher intensity than that of the untreated control and having t-test P-value less than 0.05. In addition, 363 ORFs, having statistically different intensities from that of the control with the t-test P-value less than 0.01, were recognized as induced genes. On the other hand, 73 ORFs, having two times lower intensity than that of the untreated control and having t-test P-value less than 0.05, were recognized as repressed genes. Similarly, 471 ORFs having statistically different intensities from the control with the t-test P-value less than 0.01 were also recognized as repressed genes.

From the Oligo DNA microarray, we obtained 5,869 ORFs exhibiting intensities over the cut-off value at least in one experiment. Among these ORFs, 113 ORFs showed more than two times higher intensity than that of the untreated control and having t-test P-value less than 0.05. In addition, 801 ORFs, having statistically different intensities from the control with the t-test P-value less than 0.01, were recognized as induced genes. On the other hand, 41 ORFs, having two times lower intensity than that of the untreated control and having t-test P-value less than 0.05, were recognized as repressed genes. Similarly, 1123 ORFs were recognized as repressed genes whose intensities were statistically different from that of the control with the t-test P-value less than 0.01. Apparently, the number of induced and repressed genes (P < 0.5) were higher for the ORF DNA microarray and the number of statistically significant (P < 0.01) induced and repressed genes were higher for the Oligo DNA microarray. These differences might arise from the different numbers of data collected from the two microarrays.

Table 1 lists the highly induced genes according to their average induction values obtained from the ORF and Oligo DNA microarrays without any statistical selection. The most highly induced gene was *FRM2 *followed by *AADs*, *FLR1*, *OYE3*, *GRE2*, and *MET17*. The most abundantly induced genes were *AADs*. Interestingly, *AADs*, *FLR1*, *OYE3*, *GRE2*, and *MET17 *are the genes that are significantly induced by oxidative stress[10,11]. The strongly repressed genes were listed in Table 2. In contrast to the highly induced genes, there was a good agreement between the degree of repression of the repressed genes from both the ORF and Oligo DNA microarray analysis. The most strongly repressed gene was YPL095C followed by *ARO10*, *ZRT1*, *USV1*, *CWP1*, and *RPI1*.

**Figure 4:**
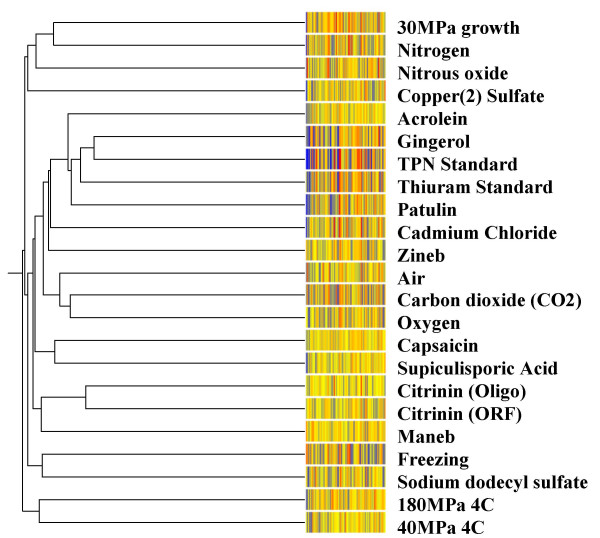
Cluster analysis of the mRNA expression profiles after the citrinin treatment. Hierarchical cluster analysis was performed using GeneSpring as described in the text.

To compare with the other stress factors, we carried out the cluster analysis of the ORF and Oligo DNA microarray expression data using the average value for each microarray. As shown in Figure 4, the expression profiles of the ORF microarray and Oligo microarray clustered together. The citrinin-induced response was very similar to that of the maneb. The citrinin-induced gene expression data did not cluster with those of the patulin, thiuram and acrolein. These results suggest that the citrinin treatment-induced response was not similar to that of the mycotoxin patulin. Thus, unlike patulin, which is known to target proteins [7,12], citrinin might not cause protein denaturation.

### Functional categogorization of citrinin induced genes

To characterize the effect of citrinin to yeast cells, the induced genes were categorized using the functional categories of MIPS. As summarized in Table 3, there were significant number of induced genes in the categories of "metabolism", "cell rescue, defense and virulence", and "energy". In addition, a high percentage of genes in these categories were found to be induced ((number of induced genes in the category/number of genes in the category) × 100). In the category of "metabolism", the subcategories of "amino acid metabolism", "nitrogen and sulfur metabolism", "metabolism of vitamins", and "secondary metabolism" were significantly induced.

In the subcategories of "amino acid metabolism" and "nitrogen and sulfur metabolism", we found that the induced genes mainly belonged to the sulfur amino acid metabolism (Table 4). Among the 25 genes listed, 21 genes can be recognized as the induced genes in at least one of the DNA microarrays. These results strongly suggest that the citrinin-treated yeast cells require methionine or glutathione. In the subcategories of "metabolism of vitamins" and "secondary metabolism", there were no groups of genes specific for vitamins and secondary metabolism, but they merely overlapped with the genes for the sulfur amino acid metabolism.

Table 5 summarized the list of the induced genes belonging to the category of "cell rescue, defense and virulence". The significantly induced genes in this category were transporters, especially the ABC transporters. Several of these transporters – such as *FLR1*, *PDR5*, *SNQ2*, *ATR1*, and *YOR1 *– are involved in multi-drug resistance, and are important for the tolerance against a broad range of organic anions [13-16]. It should be also noted that the *GTT2 *gene, which encodes the glutathione-S-transferase protein, was highly induced and the *YCF1 *gene, which codes for the vacuolar glutathione S-conjugate transporter, was also induced. The relatively significant induction of the genes in the "energy" category was due to the *AAD*s and the related genes, as these genes are categorized as the dehydrogenase (data not shown).

Citrinin was suggested to cause damages to the mitochondria. Table 6 lists the cellular localization of the induced gene products. It is clear that many of these gene products, which are localized in the mitochondria, were induced; however, the proportion of these induced genes among the total number of induced genes are not so high (Table 6, Impact). The degrees of impact values of induced genes in the mitochondria from both the microarrays were very similar to the degree of impact value of the total genes in the entries (Table 6). Although our results suggest that citrinin affected mitochondria, but we can not say that the citrinin toxicity is specific to mitochondria. In the list of highly induced genes (Table 1), the YLR346C, *GTT2*, *PDR5*, and YKL070W genes (shown in bold in Table 1) were counted as the gene products localized in the mitochondria. As these genes are also expressed in other organelles and are not specific to mitochondrial function, our results suggest that the effect of citrinin on mitochondria is true but not specific.

The functional categories of the repressed genes were also characterized (data not shown). As often seen with the stressed cells, the category of genes involved in "Protein synthesis" were significantly repressed but other significant character was not observed. The repression of the genes in the category of "Protein synthesis" can be the experimental marker, as this functional group is required for the actively growing cells, and not for the slowly growing or growth inhibited cells [17].

### Confirmation of the significantly affected genes and evaluation of both DNA microarrays

**Figure 5:**
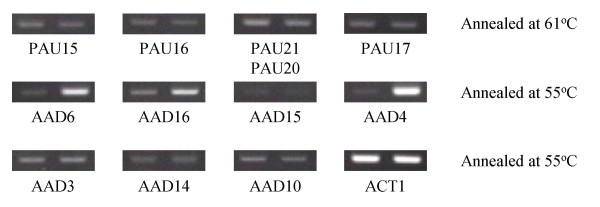
**Confirmation of gene induction by RT-PCR**. The RT-PCR analysis was performed using the primers described in Methods. Names of the genes are shown below the images.

Except the *AAD15*, *AAD10*, *AAD3*, and *PAU15*, the highly induced genes were common between the ORF DNA microarray and Oligo DNA microarray. The *AAD *genes have strong similarity to each other and this caused cross hybridization in the ORF DNA microarray [18]. Some of the highly induced *AAD *genes could cross hybridize to the ORF DNA microarray spots corresponding to the *AAD15*, *AAD10*, and *AAD3*. To confirm which *AAD *gene was really induced, we performed RT-PCR analysis. As shown in Figure 5, citrinin treatment induced the *AAD4*, *AAD6*, and *AAD16 *genes, but not the *AAD3*, *AAD10*, *AAD14*, and *AAD15 *genes. Thus, the induction of the *AAD 4, AAD6*, and *AAD16 *genes, as observed by both microarray analysis, were correct whereas the induction of the *AAD3*, *AAD10*, *AAD14*, and *AAD15 *genes in ORF DNA microarray and the induction of the *AAD14 *in Oligo DNA microarray were due to cross hybridization. We confirmed that the *AAD14 *probe has only one mismatch to the *AAD4 *ORF, and the apparent induction of the *AAD14 *was due to the cross hybridiztion to the *AAD4*. In the Oligo DNA microarray, it seems that the cross hybridization has a limit of one miss match. The *PAU15 *gene was also highly induced by citrinin treatment in Oligo DNA microarray. This gene has high similarity to other *PAU *genes, which were not induced. We, however, could not confirm the induction of the *PAU *genes by RT-PCR. Thus, the apparent induction of the *PAU15 *was most likely due to the cross hybridization with some highly induced unknown gene.

## Discussion

Mycotoxins are fungal secondary metabolites that may be toxic to all kinds of organisms. So far, a few hundreds of mycotoxins are identified and this number can increase dramatically with the development of analytical equipment. Mycotoxins are naturally occurring chemicals. The large-scale production and industrial applications of these mycotoxins are limited, because the purification of these mycotoxins are costly and inadequate. Therefore, only a few mycotoxins were studied in detail. The DNA microarray technology provides an alternative evaluation tool to examine chemical toxicity in organisms. Particularly, the yeast DNA microarray is appropriate for evaluating the action of the mycotoxin because of the less amount of toxin required in this assay and good reproducibility of the expression profile.

Citrinin is the one of the well known mycotoxins produced by *Penicillium *and *Aspergillus *family and is possibly spread all over the world [1]. The yeast-based ORF DNA microarray and Oligo DNA microarray can provide information on the possible mechanisms of toxicity and detoxification effort by yeast cells. The list of highly induced genes in citrinin-treated yeast cells (Table 1) clearly shows that the *AADs*, *OYE3*, *MET17*, and *GRE2 *genes, which are typical indicator genes for the oxidative stress [10,11], are highly induced. Thus, we can conclude that citrinin treatment causes oxidative stress. Previously, Delneli *et al. *[10] analyzed several *AAD *deletion mutants and suggested that only *AAD6 *and *AAD4 *were induced by oxidative stress. Our RT-PCR results however suggest the *AAD16 *gene is induced. Except oxidative stress, we could not find any other cell repair response. It was suggested that citrinin causes damage to the mitochondria. However, we could not confirm that citrinin specifically affects mitochondria. Mitochondria can be the source of oxidative stress. Thus, it is possible that the oxidative stress caused by citrinin could enhance the self-induced oxidative damages in mitochondria. The mycotoxin patulin produced response in yeast cells that was similar to that of the citrinin, as the oxidative stress related genes were also induced by patulin treatment [7]. In addition, the patulin treatment strongly induced the genes contributing to the protein metabolism and DNA repair, and patulin was considered as a natural mutagenic chemical [7]. However, in comparison to the patulin treatment, the citrinin treatment did not induce the genes contributing to DNA repair (Table 7). Except the oxidative stress, citrinin did not show any significant toxicity to yeast cells. The less toxicity of citrinin than the patulin was also reported in other organisms [19].

Contrast to the information concerning the mechanism of citrinin-induced toxicity, information on the detoxification mechanism was clear. The activation of the methionine and glutathione metabolisms (Table 4) strongly suggest the contribution of glutathione in the detoxification process. Moreover, strong induction of the *DTT2 *gene implies direct transfer of glutathione to citrinin. As the *PDR*s were also strongly induced (Table 5), it may be possible that the ABC transporters were involved in pumping out the citrinin-glutathione complex. Pumping out the toxin after glutathione modification is one of the main detoxification pathway used by many organism [19].

During the process of evaluating the citrinin toxicity, we also compared reproducibility of the ORF DNA microarray and Oligo DNA microarray. The Oligo DNA microarray showed higher correlation factor than the ORF DNA microarray (region B in Figure 2). This may have resulted from the cross hybridization exampled by *AAD*s. The apparent induction of the *AAD*s in the ORF DNA microarray was due to cross hybridization [7]. The Oligo DNA microarray showed less cross hybridization, as the expression levels of most of the *AAD*s obtained from this assay agreed with the RT-PCR results. However, the Oligo DNA microarray may have limits in terms of specificity, as the *AAD14 *gene, which has one mismatch with the *AAD4 *gene, was recognized as the induced gene. On the other hand, the *PAU15 *gene was not recognized as the induced gene by the ORF DNA microarray and RT-PCR, but was recognized as induced gene by the Oligo DNA microarray. If the RT-PCR results were correct, these results suggest that the high specificity may not always produce correct results. Although the Oligo DNA microarray did not completely overcome the cross hybridization in the case of single mismatch, it is still a useful tool for detecting gene expression differences between similar genes.

## Conclusion

Citrinin caused growth inhibition in yeast cells at a concentration more than 100 ppm. Under this condition, we monitored the citrinin treatment-induced response using the ORF DNA microarray and Oligo DNA microarray. Results obtained from these microarray experiments suggest that citrinin induced oxidative stress in the yeast cells. The citrinin-induced genotoxicity was less severe than that of the patulin. Thus, citrinin is a less toxic substance than patulin. The expression profiles obtained from both types of DNA microarrays were essentially similar. The reproducibility of the expression profiles were much better and the cross hybridization was less with the Oligo DNA microarray.

## Methods

### Strain, growth conditions, and citrinin treatment

*Saccharomyces cerevisiae *strain S288C (Mat alpha *SUC2 mal mel gal2 CUP1*) was grown in YPD medium (2% polypeptone, 1% yeast extract, 2% glucose) at 25°C as a pre-culture for 2–3 days. This strain was used because the ORF DNA microarray probes were produced using the S288C DNA as the template for PCR [6] and because Oligo DNA microarray probes were designed based on the DNA sequence of this strain [20]. Citrinin was purchased from MP Biochemicals (Irvine, CA, USA) and was dissolved in DMSO (Dimethyl sulfoxide) to prepare a stock solution of 20000 ppm. To monitor the dose response of citrinin to yeast cells, the stock solution was added directly to the YPD medium containing the yeast cells such that they were diluted more than 100-fold. For the DNA microarray analysis, yeast cultures in YPD were diluted and grown overnight to an optical density (OD660) of 1.0. The citrinin stock solution was added to the cultures and yeast cells were allowed to grow for an additional 2 h. For the control cells, the same volume of DMSO was added to the yeast culture and this was incubated for 2 h. Cells were harvested by centrifugation and stored at -80°C until used.

### DNA microarray analysis

DNA microarray analysis was carried out on three independent cultures and total RNA was isolated by the hot-phenol method as described previously [21].

For the ORF type DNA microarray, yeast DNA microarray Ver. 2.0 (DNA Chip Research, Inc., Yokohama, Japan) was used and the hybridization was performed using the dual color method. The Cy3- or Cy5-labeled cDNA pools were synthesized by CyScribe First-Strand cDNA Labeling Kit (GE Healthcare UK Ltd., Buckinghamshire, England). On this microarray, a total of 6,037 kinds of amplified ORFs with 200–8,000 bp length (0.1–0.5 ng) were spotted. The Cy3- or Cy5-labeled aRNA mixed pools were hybridized for 24–36 h at 65°C. The details of our conditions for the microarray procedure and validation studies were previously described [6-8,21,22].

For the Oligo DNA microarray, 3D-Gene Yeast Oligo Chip 6K (Toray Industries Inc., Tokyo, Japan/DNA Chip Research, Inc., Yokohama, Japan) was used. For efficient hybridization, this microarray has 3-dimensions that is constructed with a well as the space between the probes and cylinder-stems with 30-mer oligonucleotide probes on the top. Total RNA was labeled with Cy3- or Cy5- using the Amino Allyl MessageAMP II aRNA Amplificatin Kit (Applied Biosystems, CA, U.S.A.). The Cy3- or Cy5-labeled aRNA pools and hybridization buffer containing micro beads were mixed, and hybridized for 16 h. The hybridization was performed using the supplier's protocols.

### Data analysis

Detected signals for each ORF were normalized by the intensity dependent (LOWESS) methods [23]. The cutoff values were the intensity of the background average plus 2SD. Genes were characterized for function according to the functional categories established by MIPS [24] and the SGD [25]. The data obtained in this experiment have been assigned accession number GSE6118 in the Gene Expression Omnibus Database [26].

Hierarchical cluster analysis was performed using the GeneSpring ver. 7.3.1 software (Silicon Genetics, CA, USA). The clustering algorithm arranges conditions according to their similarity in the expression profiles across all conditions, such that conditions with similar patterns are clustered together as in a taxonomic tree. Data from 3874 genes were used for the calculation. These 3874 genes were selected on the basis of having previously exhibited higher than average intensities in another trial [21].

### RT-PCR

A reverse transcriptase-polymerase chain reaction (RT-PCR) was carried out to confirm the result of the microarray experiments for the genes showing different patterns of expression between the ORF type microarray and the oligo probe microarray. The primers for the *AADs *were described previously [7]. The primers for the *PAU*s are:

*PAU15 *(YIR041W),

CTTGTTTCAAGCAGCTCATCCAAGT and ATGGAATCTCATTCGTAAAGGCATG; *PAU16*(YKL224C),

CTTGTTTCAAGCAGCTCATCCAAGT and CATATTCATAAAATGCTTCACG; *PAU21/22 *(YOR394W, YPL282C),

TACCAGATTGAGACCGGCTATC and TACTCCACAAACACTGTTATTG; and

*PAU17 *(YLL025W),

GAGCTCATTTGGCTGAATACTATATG and TGCAGATAGAGCGCTGGAGATG. Total RNA prepared for the microarray analysis was used as template for the RT-PCR experiments. Reverse transcriptase reaction was performed using the StrataScript First-Strand Synthesis System (STRATAGENE, CA, USA). The cDNA mixture was diluted 20 times, and 2 μl of the diluted solution was used for a 20 μl PCR reaction using the TaKaRa Ex Taq HS (TaKaRa, Shiga, Japan). Annealing temperature was originally set at 55°C. However, the *PAU*s showed multiple bands at 55°C and annealing temperature was increased to 61°C. Each amplification reaction was resolved on a 2% agarose gel and the DNA bands were visualized with EtBr staining.

## Abbreviations

ORF: open reading frame

Oligo: oligo-nucleotide

MIPS: Munich Information Center for Protein Sequences

SGD: Yeast Genome Database

DMSO: Dimethyl sulfoxide

RT-PCR : reverse transcriptase-polymerase chain reaction

## Authors' contributions

HI planned and designed the study and wrote the main draft of the manuscript. EK analyzed the DNA microarray results and performed the RT-PCR experiments. YS performed the ORF DNA microarray experiments. YU and YI performed the Oligo DNA microarray experiments. HN and YK analyzed the Oligo DNA microarray results and contribute on the cross hybiridization search based on the ligo nucleotide on the microarray. HH contributed the selection of mycotoxin and planned the experiments. YI has the responsibility for the budget supporting the most part of this work and planned and performed the mycotoxin experiments. All authors read and approved the final manuscript.

## Figures and Tables

**Table 1:** List of highly induced genes by the citrinin treatment

			ORF-Array		Oligo-Array		
					
Systematic Name	Common Name	Average (Fold)	Fold	t-test P-Value	Fold	t-test P-Value	MIPS_Description
YCL026C-A	FRM2	104.0	162.4	0.002	45.7	0.000	Involved in fatty acid regulation
YFL057C	AAD16	63.5	86.1	0.003	40.8	0.000	Aryl-alcohol dehydrogenase
YFL056C	AAD6	47.0	39.8	NA*	54.2	0.000	Putative aryl-alcohol dehydrogenase,
YDL243C	AAD4	46.3	53.4	0.000	39.2	0.000	Aryl-Alcohol Dehydrogenase
YBR008C	FLR1	33.6	37.9	0.000	29.4	0.000	Putative H+ antiporter involved in multidrug resistance
YPL171C	OYE3	29.9	31.9	0.001	27.8	0.000	NAPDH dehydrogenase (old yellow enzyme), isoform 3
YOL165C	AAD15	26.6	51.3	0.000	1.9	0.000	Putative aryl alcohol dehydrogenase
YIR041W	PAU15	23.6	1.7	0.159	45.3	0.000	Similarity to members of the Srp1p/Tip1p family
YJR155W	AAD10	22.3	43.7	0.000	1.0	0.858	Putative aryl-alcohol dehydrogenase
YNL331C	AAD14	22.3	21.5	0.001	23.1	0.000	Putative aryl-alcohol dehydrogenase
YLR346C**		22.3	22.9	0.002	21.7	0.000	Protein of unknown function localised to mitochondria
YOL151W	GRE2	19.5	18.3	0.000	20.7	0.000	Methylglyoxal reductase (NADPH-dependent)
YCR107W	AAD3	15.0	28.4	0.000	1.6	0.000	Aryl-alcohol dehydrogenase
YLR303W	MET17	14.7	12.3	0.000	17.1	0.000	O-acetylhomoserine sulfhydrylase
YLL056C		13.6	16.5	0.000	10.7	0.000	Weak similarity to Y. pseudotuberculosis epimerase
YLL060C**	GTT2	13.2	13.1	0.000	13.3	0.000	Glutathione S-transferase
YOR153W*	PDR5	12.5	16.3	0.000	8.8	0.000	ABC transporter involved in multidrug resistance
YGR213C	RTA1	12.3	9.7	0.001	15.0	0.000	Integral membrane protein
YOR049C		12.2	11.1	0.001	13.3	0.000	Putative integral membrane transporter
YKR076W	ECM4	11.2	11.7	0.000	10.7	0.000	Involved in cell wall biogenesis and architecture
YML131W		10.7	9.2	0.000	12.2	0.000	Putative hydroxydehydrogenase
YKL070W**		10.7	9.1	0.020	12.3	0.000	Similarity to B. subtilis transcriptional regulatory protein
YIL167W		9.3	9.0	0.005	9.5	0.000	Serine dehydratase

* NA, Not applicable (experiment was either performed less than three times or the data was not valuable

** Names indicated in bold means the genes whose products are localized in the mitochondria

**Table 2:** List of strongly repressed genes by the citrinin treatment

			ORF-Array		Oligo-Array		
					
Systematic Name	Common Name	Average (Fold)	Fold	t-test P-value	Fold	t-test P-value	MIPS_Description
YPL095C		0.19	0.19	0.002	0.18	0.000	Hypothetical ORF
YDR380W	ARO10	0.29	0.36	0.038	0.21	0.000	Phenylpyruvate decarboxylase
YGL255W	ZRT1	0.33	0.36	0.015	0.30	0.000	High-affinity zinc transporter
YKL096W	CWP1	0.35	0.38	0.000	0.32	0.000	Cell wall mannoprotein
YIL119C	RPI1	0.37	0.28	0.028	0.46	0.000	Putative transcriptional regulator
YHL028W	WSC4	0.39	0.51	0.033	0.28	0.000	Cell wall integrity and stress response
YHR137W	ARO9	0.40	0.37	0.001	0.43	0.000	Aromatic aminotransferase
YPR194C	OPT2	0.40	0.46	0.020	0.35	0.000	Oligopeptide transporter
YMR120C	ADE17	0.41	0.41	0.007	0.41	0.000	Enzyme of 'de novo' purine biosynthesis
YAR015W	ADE1	0.42	0.31	0.002	0.54	0.000	SAICAR synthetase
YMR011W	HXT2	0.43	0.41	0.003	0.44	0.000	High-affinity glucose transporter
YPR160W	GPH1	0.44	0.41	0.018	0.47	0.000	Non-essential glycogen phosphorylase
YPL092W	SSU1	0.44	0.41	0.009	0.48	0.000	Plasma membrane sulfite pump
YBL098W		0.45	0.42	0.002	0.47	0.000	Kynurenine 3-mono oxygenase
YFR015C	GSY1	0.45	0.48	0.024	0.42	0.000	Glycogen synthase
YOR315W		0.45	0.41	0.007	0.50	0.000	Protein of unknown function,
YDL227C	HO	0.45	0.49	0.048	0.42	0.000	Site-specific endonuclease

**Table 3:** Contribution of induced genes to functional categories

		ORF DNA microarray				OligoDNA microarray			
			
		F > 2 & P < 0.05*		P < 0.01*		F > 2 & P < 0.05*		P < 0.01*	
					
Category subcategory	Total number in category	Number	%*	Number	%	Number	%	Number	%
Metabolism	1521	54	3.6	103	6.8	51	2.4	266	17.0
amino acid metabolism	243	20	8.2	33	13.6	25	10.3	81	33.3
nitrogen and sulfur metabolism	96	9	9.4	15	15.6	11	11.5	39	40.6
nucleotide metabolism	227	6	2.6	12	5.3	2	0.9	22	9.7
phosphate metabolism	414	7	1.7	15	3.6	6	1.4	55	13.3
C-compound and carbohydrate metabolism	504	18	3.6	36	7.1	19	3.8	86	17.1
lipid, fatty acid and isoprenoid metabolism	272	7	2.6	20	7.4	5	1.8	30	11.0
metabolism of vitamins,	163	11	6.7	16	9.8	9	5.5	40	24.5
secondary metabolism	77	4	5.2	7	9.1	4	5.2	19	24.7
ENERGY	365	17	4.7	34	9.3	15	4.1	59	16.2
CELL CYCLE AND DNA PROCESSING	1001	9	0.9	37	3.7	4	0.4	119	11.9
TRANSCRIPTION	1063	10	0.9	39	3.7	9	0.8	87	8.2
PROTEIN SYNTHESIS	476	3	0.6	20	4.2	1	0.2	8	1.7
PROTEIN FATE (folding, modification, destination)	1137	23	2.0	65	5.7	8	0.7	159	14.0
PROTEIN WITH BINDING FUNCTION	1034	22	2.1	54	5.2	15	1.5	134	13.0
PROTEIN ACTIVITY REGULATION	238	2	0.8	6	2.5	1	0.4	23	9.7
CELLULAR TRANSPORT	1031	33	3.2	74	7.2	18	1.7	131	12.7
CELLULAR COMMUNICATION	234	1	0.4	4	1.7	1	0.4	28	12.0
CELL RESCUE, DEFENSE AND VIRULENCE	548	31	5.7	47	8.6	28	5.1	118	21.5
INTERACTION WITH THE CELLULAR ENVIRONMEN	458	16	3.5	28	6.1	9	2.0	71	15.5
INTERACTION WITH THE ENVIRONMENT	5	0	0.0	0	0.0	0	0.0	1	20.0
TRANSPOSABLE ELEMENTS	124	1	0.8	1	0.8	0	0.0	5	4.0
DEVELOPMENT (Systemic)	70	1	1.4	4	5.7	0	0.0	7	10.0
BIOGENESIS OF CELLULAR COMPONENTS	854	11	1.3	31	3.6	6	0.7	95	11.1
CELL TYPE DIFFERENTIATION	449	3	0.7	18	4.0	2	0.4	48	10.7
UNCLASSIFIED PROTEINS	2038	37	1.8	74	3.6	23	1.1	163	8.0
Total		155		363		113		801	

**Table 4:** Glutathione and methionine metabolism related genes are induced by the citrinin treatment

			ORF-Array		Oligo-Array		
					
Systematic Name	Common Name	Average (Fold)	Fold	t-test P-value	Fold	t-test P-value	Description
YKR069W	MET1	2.6	2.3	0.002	2.9	0.000	siroheme synthase
YFR030W	MET10	3.2	1.8	0.025	4.6	0.000	sulfite reductase flavin-binding subunit
YKL001C	MET14	5.2	5.3	0.000	5.1	0.000	ATP adenosine-5^-phosphosulfate 3^-phosphotransferase
YPR167C	MET16	5.1	5.6	0.001	4.6	0.000	3^-phosphoadenylylsulfate reductase
YLR303W	MET17	14.7	12.3	0.000	17.1	0.000	O-acetylhomoserine sulfhydrylase
YNL277W	MET2	2.9	2.1	0.022	3.8	0.000	homoserine O-acetyltransferase
YOL064C	MET22	1.7	1.8	0.000	1.7	0.000	protein ser/thr phosphatase
YIR017C	MET28	4.7	2.0	0.198	7.5	0.000	transcriptional activator of sulfur amino acid metabolism
YJR010W	MET3	8.9	6.0	0.000	11.8	0.000	sulfate adenylyltransferase
YIL046W	MET30	1.1	1.2	0.203	1.1	0.147	involved in regulation of sulfur assimilation genes
YPL038W	MET31	1.0	1.2	0.151	0.9	0.004	transcriptional regulator of sulfur amino acid metabolism
YDR253C	MET32	2.7	2.2	0.004	3.3	0.000	transcriptional regulator of sulfur amino acid metabolism
YNL103W	MET4	1.0	0.7	0.102	1.2	0.001	transcriptional activator of sulfur metabolism
YER091C	MET6	2.7	2.6	0.001	2.7	0.000	homocysteine methyltransferase
YBR213W	MET8	1.4	1.2	0.460	1.6	0.000	siroheme synthase
YAL012W	CYS3	2.0	1.9	0.009	2.0	0.000	cystathionine gamma-lyase
YGR155W	CYS4	1.9	2.4	0.062	1.5	0.000	cystathionine beta-synthase
YJL101C	GSH1	2.4	2.2	0.000	2.7	0.000	glutamate – cysteine ligase
YOL049W	GSH2	1.1	0.9	0.064	1.2	0.000	Glutathione synthetase
YLR180W	SAM1	1.4	1.6	0.023	1.3	0.000	S-adenosylmethionine synthetase 1
YDR502C	SAM2	1.6	1.5	0.000	1.6	0.000	S-adenosylmethionine synthetase 2
YPL274W	SAM3	1.2	1.3	0.015	1.1	0.124	S-adenosylmethionine permease
YPL273W	SAM4	0.9	0.8	0.010	1.0	0.008	AdoMet-homocysteine methyltransferase
YJR130C	STR2	1.4	1.2	NA	1.6	0.000	Cystathionine gamma-synthase
YGL184C	STR3	2.1	1.4	0.367	2.9	0.001	cystathionine beta-lyase

* NA, Not applicable (experiment was either performed less than three times or the data was not valuable

**Table 5:** List of highly induced genes in the category of "CELL RESCUE, DEFENSE AND VIRULENCE"

			ORF-Array		Oligo-Array		
					
Systematic Name	Common Name	Average (Fold)	Fold	t-test P-value	Fold	t-test P-value	Description
YBR008C	FLR1	33.6	37.9	0.000	29.4	0.000	Plasma membrane multidrug **transporter**
YOL151W	GRE2	19.5	18.3	0.000	20.7	0.000	NADPH-dependent methylglyoxal reductase
YLL060C	GTT2	13.2	13.1	0.000	13.3	0.000	Glutathione S-transferase
YOR153W	PDR5	12.5	16.3	0.000	8.8	0.000	Short-lived membrane ABC **transporter**
YGR213C	RTA1	12.3	9.7	0.001	15.0	0.000	involved in 7-aminocholesterol resistance
YHR048W		5.8	3.4	0.003	8.2	0.000	Hypothetical ORF
YDR011W	SNQ2	5.2	6.6	0.000	3.8	0.000	ABC **transporter**
YML116W	ATR1	5.2	5.5	0.000	4.8	0.000	Multidrug efflux pump of the major facilitator superfamily
YGR281W	YOR1	4.6	4.7	0.000	4.5	0.000	ABC **transporter**
YNL231C	PDR16	3.8	3.4	0.000	4.3	0.000	Phosphatidylinositol transfer protein
YHL040C	ARN1	3.8	3.1	0.002	4.5	0.000	Member of the ARN family of **transporters**
YNL160W	YGP1	3.2	2.9	0.000	3.5	0.000	May be involved in cellular adaptations prior to stationary pha
YMR038C	LYS7	3.1	3.3	0.000	3.0	0.000	Copper chaperone for superoxide dismutase Sod1p
YGR209C	TRX2	3.0	3.1	0.025	3.0	0.000	Thioredoxin
YMR173W	DDR48	2.9	3.0	0.003	2.8	0.000	DNA damage-responsive protein
YHR136C	SPL2	2.8	3.5	0.000	2.2	0.000	Protein with similarity to cyclin-dependent kinase inhibitors
YDR533C		2.8	3.0	0.000	2.6	0.000	Possible chaperone and cysteine protease
YER042W	MXR1	2.6	2.2	0.001	2.9	0.000	Peptide methionine sulfoxide reductase
YBL064C		2.5	3.0	0.000	2.1	0.000	Mitochondrial peroxiredoxin with thioredoxin peroxidase
YER185W		2.5	2.8	0.003	2.3	0.000	Hypothetical ORF
YDR135C	**YCF1**	2.5	ND*		2.5	0.000	Vacuolar glutathione S-conjugate **transporter**
YDR032C	PST2	2.5	2.6	0.005	2.4	0.000	Similarity to members of a family of flavodoxin-like proteins
YJL101C	GSH1	2.4	2.2	0.000	2.7	0.000	Gamma glutamylcysteine synthetase

* ND, Not determined

**Table 6:** Localization of the citrinin-induced gene products

			ORF DNA microarray				OligoDNA microarray			
				
	Entries		F > 2 & P < 0.05		P < 0.01		F > 2 & P < 0.05		P < 0.01	
					
Localization	Number	Impact*	Number	Impact	Number	Impact	Number	Impact	Number	Impact
extracellular	54	1.0	2	1.4	6	1.8	4	3.9	10	1.4
bud	149	2.9	3	2.2	5	1.5	0	0.0	13	1.8
cell wall	42	0.8	1	0.7	4	1.2	3	2.9	7	1.0
cell periphery	216	4.1	11	8.0	20	5.9	8	7.8	30	4.2
plasma membrane	186	3.6	8	5.8	18	5.3	5	4.9	29	4.1
integral membrane/endomembranes	176	3.4	10	7.2	14	4.2	7	6.9	23	3.2
cytoplasm	2906	55.8	94	68.1	191	56.7	76	74.5	449	63.2
cytoskeleton	204	3.9	3	2.2	5	1.5	2	2.0	25	3.5
ER	557	10.7	13	9.4	57	16.9	8	7.8	92	13.0
golgi	132	2.5	2	1.4	8	2.4	1	1.0	16	2.3
transport vesicles	139	2.7	2	1.4	6	1.8	0	0.0	13	1.8
nucleus	2157	41.4	49	35.5	129	38.3	35	34.3	304	42.8
mitochondria	1056	20.3	33	23.9	77	22.8	21	20.6	149	21.0
peroxisome	52	1.0	2	1.4	3	0.9	0	0.0	5	0.7
endosome	57	1.1	1	0.7	5	1.5	1	1.0	10	1.4
vacuole	280	5.4	14	10.1	27	8.0	8	7.8	47	6.6
microsomes	5	0.1	0	0.0	0	0.0	0	0.0	1	0.1
lipid particles	27	0.5	2	1.4	4	1.2	1	1.0	3	0.4
punctate composite	141	2.7	5	3.6	9	2.7	4	3.9	15	2.1
ambiguous	237	4.5	6	4.3	18	5.3	4	3.9	37	5.2
KNOWN LOCALIZATION	5209	100	138	100	337	100	102	100	710	100
UNKNOWN LOCALIZATION	1516		17		26		11		91	

**Table 7:** Comparison of the patulin – and citrinin-induced genes contributing to DNA repair

	Fold Induction			
			
Systematic Name	Patulin	Citrinin	Common Name	MIPS_Description
YDL059C	5.7	1.8	RAD59	Recombination and DNA repair protein
YGL163C	5.3	1.0	RAD54	DNA-dependent ATPase of the Snf2p family
YGR209C	4.4	3.0	TRX2	Thioredoxin II
YDR092W	4.0	1.2	UBC13	E2 ubiquitin-conjugating enzyme
YER142C	3.9	1.5	MAG1	3-methyladenine DNA glycosylase
YHL024W	3.7	1.1	RIM4	No sporulation
YFL014W	3.7	0.9	HSP12	Heat shock protein
YPR193C	3.7	1.2	HPA2	Histone and other Protein Acetyltransferase
YKL145W	3.5	1.3	RPT1	26S proteasome regulatory subunit
YMR173W	3.4	2.9	DDR48	Heat shock protein
YAR007C	3.4	1.0	RFA1	DNA replication factor A, 69 KD subunit
YPL194W	3.1	1.1	DDC1	DNA damage checkpoint protein
YLR043C	3.0	1.4	TRX1	Thioredoxin I
YOR023C	2.9	1.0	AHC1	Component of the ADA histone acetyltransferase comple
YEL037C	2.8	1.1	RAD23	Nucleotide excision repair protein (ubiquitin-like protein)
YMR302C	2.8	0.9	PRP12	Involved in early maturation of pre-rRNA
YNL312W	2.7	1.2	RFA2	DNA replication factor A, 36 kDa subunit
YIL143C	2.7	1.1	SSL2	DNA helicase
YJR052W	2.6	1.1	RAD7	Nucleotide excision repair protein
YOL068C	2.4	0.7	HST1	Silencing protein
YGR231C	2.4	1.0	PHB2	Prohibitin
YPR023C	2.3	1.2	EAF3	Esa1p-associated factor
YML032C	2.3	1.1	RAD52	Recombination and DNA repair protein
YIR025W	2.2	1.2	MND2	Subunit of anaphase-promoting complex
YGL201C	2.1	1.0	MCM6	Involved in replication
YMR201C	2.1	0.9	RAD14	Nucleotide excision repair protein
YNL250W	2.0	1.2	RAD50	DNA repair protein
YCR086W	2.0	1.5	CSM1	Involved in nuclear migration
